# Experiences and perceptions of the theory‐practice gap in nursing in a resource‐constrained setting: A qualitative description study

**DOI:** 10.1002/nop2.188

**Published:** 2018-07-19

**Authors:** David Abdulai Salifu, Janet Gross, Mohammed Awal Salifu, Jerry PK Ninnoni

**Affiliations:** ^1^ Principal Health Tutor, Ministry of Health, Nurses' Training College Damongo Ghana; ^2^ Professor Emerita Morehead State University KY USA; ^3^ Global Health Services Partnership US Peace Corps Liberia; ^4^ Senior Health Tutor, Ministry of Health Nurses’ Training College Damongo Ghana; ^5^ Department of Mental Health, School of Nursing and Midwifery, College of Health and Allied Sciences University of Cape Coast Cape Coast Ghana

**Keywords:** community of learning, nursing education, theory‐practice gap, qualitative description

## Abstract

**Aim:**

To describe experiences and perceptions of theory‐practice gap in nursing in a resource‐constrained setting. Theory‐practice gap is extensively discussed and studied in some parts of the world. Interventions to bridge the theory‐practice gap have been varied and depend on an understanding of the contextual healthcare environment. Experiences and perceptions of the theory‐practice gap in a resource‐constrained setting have not been comprehensively described.

**Design:**

A qualitative description methodology was used.

**Methods:**

Maximum variation sampling based on role in the events of theory‐practice gap was used to recruit student nurses, nurse faculty and clinicians from two study sites for focus group discussions. Data were analysed using conventional content analysis.

**Results:**

Five themes were identified: system inadequacies; resource constraints; challenges of the clinical learning environment; clinical placement and supervision; and nurse faculty factors. Systems inadequacy and resource constraints formed the spine of the challenges contributing to the theory‐practice gap in the research setting.

**What is already known about this topic**

- Theory‐practice gap is a reverberative theme in nursing education and practice with the potential to impede advances in nursing practice and patient safety
- The professional and bureaucratic work conflict has been proposed as an explanatory model for the existence of the theory practice gap

**What this paper adds**

- Poor support for student learning in the clinical learning environment can occur when the community of learning is poorly constituted
- Varying levels of the TPG exist in various settings based on the degree of mal‐alignment between learning outcomes, learning activities and the learning environment (consisting of the community of learning)

## 1 INTRODUCTION

Globally, the goal of nursing education is to ensure professional clinical competencies and to enhance the delivery of safe, quality nursing care (Forsberg, Georg, Ziegert, & Fors, 2011; Tseng, et al., 2011; World Health Organisation [WHO], 2007).With nursing as a practice‐oriented discipline, education not only involves theoretical content discussed in classrooms but also requires sufficient clinical placements to allow for skills development and the application of theoretical content to practice. This can only be achieved by ensuring that nursing students apply what they have learnt in the classroom and simulation laboratories to real‐world situations (Lauder, Sharkey, & Booth, 2004). The theory‐practice gap (TPG) has been a frequently identified construct in nursing education and practice because the integration of theoretical content in practice does not usually occur smoothly (Bendal, 2006; Lee, 1996). The frustrations and difficulties associated with the TPG are largely experienced by student nurses and newly qualified nurses and can have an adverse impact on their socialization into the professional role (Jamshidi, 2012; Maben, Latter, & Clark, 2006; Monaghan, 2015; Wynaden, Orb, McGown, & Dowie, 2000). The reality shock, introduced by a wide TPG, is considered as a major cause of the low job satisfaction and high job attrition rates among newly qualified nurses (Al Awaisi, Cooke, & Pryjmachuk, 2015; Yang, Chao, Lai, Chen, Shih, & Chiu, 2013). The TPG has also been cited as a contributory factor in medication errors (Gregory, Guse, Davidson, Davis, & Russel, 2009; Jones & Treiber, 2010) and reduced use of physical assessment skills among nurses (Secrest, Norwood, & Dumont, 2005). Clearly, the existence of the TPG has the potential to influence the quality of nursing care and patient outcomes. This article describes the experiences and perceptions of the TPG in a resource‐limited setting.

## 2 BACKGROUND

Various explanations have been proffered for the existence of the TPG in nursing education and practice. Landers (2000) discussed these reasons under two main arms; nursing theory factors and issues related to clinical settings. She argued that not only does a TPG exist but there is also a theory‐theory gap between nurse scholars and practitioners which contributes to the TPG. Formal theories proposed by nurse theorists often seek to offer generalizable representations of nursing practice which, are often meaningless because it is almost impossible to accurately describe and theoretically represent the complex and evolving clinical environment (Ashworth & Longmate, 1993; Landers, 2000). Informal theories, thought of as practically relevant, risk becoming inert as the resultant knowledge may not be adequately understood by students to have any practical utility (Hislop, Ingles, Cope, Stoddart, & McIntosh, 1996). Some authors have also attempted to relate the existence of the TPG to the inability of nursing teachers to assume a central role in clinical teaching and learning (Gerrish, 1992) and the shift in nursing education from the traditional hospital‐based approach to higher education institutions (Hewison & Wildman, 1996).

The ethos of the clinical setting plays a decisive role in students’ clinical learning and theory‐practice integration (Landers, 2000). It has long been suggested that high workloads, a routine and monotonous approach to care and a strained clinical learning environment does little to promote clinical learning and can thwart initiative and creativity (Craddock, 1993; Ogiers, 1989). Maben et al. (2006) described the impact of the clinical environment on the realization of the ideals and values taught in nursing education programmes as the professional‐bureaucratic work conflict. While it is evident that support for students and newly qualified nurses in the clinical environment is a prerequisite for theory‐practice integration (Killam & Heerschap, 2013; Monaghan, 2015), there seems to be no widely acknowledged approach to supporting student learning in the clinical environment.

Approaches to supporting student learning have not fully used the mix of personnel interacting with students and assisting in clinical learning activities. Some of these approaches use either nurse faculty (link teachers and clinical faculty), clinicians (preceptorship) or both (lecturer‐practitioners) (Landers, 2000). The main challenge with these approaches appears to be the lack of support from other personnel in the clinical learning environment who have not been outwardly assigned the responsibility of supporting student learning (Corlett, 2000; Dahlke & Hannesson, 2016; Killam & Heerschap, 2013). Botma, Rensburg, Coetzee, & Heyns (2015) suggested the establishment of a community of learning consisting of students, facilitators and clinicians guided by clear learning outcomes, as a prerequisite for theory‐practice integration. Experiences of the TPG may be varied due to differences in pedagogical approaches and healthcare environments between more technologically advanced countries and resource‐constrained settings.

## 3 THE STUDY

### 3.1 Aim

The aim of the study was to describe the experiences and perceptions of strategic stakeholders in nursing education and practice of events associated with TPG in a resource‐constrained setting.

### 3.2 Design

A qualitative description methodology of the naturalistic paradigm was adopted to describe the common, everyday experiences and perceptions of events contributing to the phenomenon of theory practice gap in a way specifically relevant for improving practice, education, research and policy decisions (Sandelowski, 2000).

### 3.3 Context

The education of nurses in the resource‐constrained setting where the research took place primarily occurred in the diploma‐awarding Nursing Training Colleges (NTCs), the majority of which were affiliated with a hospital (Bell, Rominski, Bam, Donkor, & Lori, 2013; Talley, 2006). The numbers of higher education institutions providing baccalaureate level nursing education in the research setting had increased in the past decade although NTCs still predominate. It has been noted that the infrastructure required for effective teaching and learning in the NTCs and other institutions of higher learning has been inadequate and poorly equipped to meet minimum requirements for quality teaching and learning (Bell et al., 2013). The existing higher education institutions also lacked adequate numbers of qualified nursing faculty, with ongoing recruitment drives stifled by the unavailability of highly qualified nurse faculty (Bell et al., 2013). Additionally, the lack of qualified nursing faculty limited the number of higher educational institutions offering postgraduate nursing education and bringing to question the quality of nurse educators at the various NTCs.

### 3.4 The study sites

The study took place at two sites situated in the poorest region of the country with a poverty level of 50.4%, representing 1.3 million poor individuals (Cooke, Hague, & McKay, 2016). Site A was a teaching hospital and the main referral point in the region. Site B was a university which had been in existence for more than three decades but only started offering a baccalaureate nursing programme about a decade ago. The Nursing Department of site B had an average class size of 176 and an average student population of 704 at the time of the study. The Department depended on the services of adjunct faculty to help augment the staff shortage. The Department was faced with a myriad of challenges. Educational resources were limited. Nursing faculty and students do not have access to the wealth of data available online to benefit from the learning process. The school does not have an electronic library system and their wireless connection was erratic. The Department does not have its own library and the simulation room was poorly supplied and some equipment was outdated. Disposable nursing supplies were simply inadequate or not available to facilitate teaching.

Students at site B were mainly assigned clinical placements at site A but also used other smaller clinical placement areas in the region and across the country. Typically, only a few of the clinicians acting as preceptors in site A possessed a postgraduate level education in nursing and did so with minimal to no training for the role.

### 3.5 Sample/participants

Maximum variation purposive sampling was used to recruit nursing students and nurse faculty from site B and clinicians from site A. This approach to sampling helped to identify and describe not only the particularly important experiences and perceptions common to the diverse groups of participants but also those peculiar to each group (Patton, 1990). A focal person responsible for dissemination of the research agenda was identified at each site. Letters were sent to the head of the department of nursing at site B and director of nursing service at site A, detailing the inclusion criteria for participant selection (Table 1), venue and dates for focus group discussions and contacts of the focal person a month prior to data collection. Potential participants were identified via the focal person and were sent weekly text messages as reminders.

**Table 1:** Inclusion/exclusion criteria

Inclusion criteria	Exclusion criteria
Clinicians with a minimum of a bachelor's degree in nursing and at least three years clinical working experience and acting as a preceptor, or a clinician with a minimum of diploma in nursing and five years working experience and acting as a preceptor in site A. Only full‐time nurse faculty of site B with at least three years teaching experience and a minimum qualification of a master's degree. Level 400 postsecondary nursing students of site B	The study excluded nurse faculty, clinicians and nursing students who did not meet the inclusion criteria.

### 3.6 Data collection

Separate and homogenous focus group discussions were held with each of the three categories of participants with the aid of a topic guide (Table 2). Homogenously constituted focus groups allowed participants to hear diverse views and freely add their own perspectives and insights as the search for the central themes of the phenomenon unfolded (Bowling, 2014). Two researchers were involved in facilitating focus group discussions; one acting as a moderator and the other as an observer responsible for issuing consent forms, noting non‐verbal cues and time keeping. The number of discussion sessions held depended on reaching data saturation, that is, when no new themes were emerging from the participants and the data were repetitive (Streubert & Carpenter, 2011). All discussions were held in English language, tape recorded and copied to a pass‐worded computer accessible only to the research team. Data were collected from March to June 2016.

**Table 2:** Topic guide for focus group discussion on theory‐practice gap (TPG)

1. Tell me all that you know about nursing education in the study setting with regards to equipping nursing students with the necessary skills to practice.
2. Tell me all that you know about the TPG
3. Could you describe situations where you have identified TPG?
4. In your opinion, what do you think brings about TPG?
5. What in your opinion are the positive implications of TPG in nursing?
6. What in your opinion are the negative implications of TPG in nursing?
7. How does the gap affect you as a professional?
8. Tell me what the profession stands to gain in bridging the TPG
9. What can we do to influence changes, if any, in this gap?

### 3.7 Ethical considerations

Ethical approval for the study was granted by University Institutional Review Board. Approval was also given by both sites A and B prior to data collection. Participants were comprehensively informed about the aims and procedures of the study before a written informed consent was obtained from each of the participants. The confidentiality and anonymity of participants were ensured by assigning a code to each participant; known only to the participant and researchers. The study process did not entail any manipulations or potentially harmful effects on participants.

### 3.8 Data analysis

Data from focus group discussions were analysed using conventional content analysis to allow for the sub‐themes and cluster themes to emerge exclusively from the data (Hsieh & Shanon, 2005). Audiotapes of focus group data were translated verbatim and both tapes and transcripts listened to and read respectively several times by two investigators to allow for immersion and appreciation of the whole data. Transcripts were then read word‐by‐word and notes made of words or phrases from the text that represented underlying thoughts or concepts. This approach continued until an overarching label for a sub‐theme representative of all the initial concepts or thoughts emerged. The two investigators each independently arrived at a labelling scheme but then discussed and agreed on a final labelling scheme. Data that did not fit into the labelling system were discussed to reach a consensus. New labels were then developed. All data within a sub‐theme were examined to ensure a fit between the data and the sub‐theme. Sub‐themes were then grouped into cluster themes and further into emergent themes by agreement of the two investigators. The analytical process was reviewed by a third researcher comparing codes and transcripts to enhance trustworthiness.

### 3.9 Rigour

Data sources for this study were triangulated to ensure a holistic coverage of the phenomenon from perceptions of stakeholders with extensive experience of the events of TPG. The topic guide for focus group discussions was reviewed by two expert nurse educationists and piloted with student nurses, nurse faculty and clinicians at two sites similar to the study sites but in a different region. Minor revisions were made to wording of the topic guide to enhance clarity. Data analysis was also triangulated, and the analytical process peer reviewed by one of the co‐authors.

## 4 FINDINGS

A total of 32 participants were recruited; nurse faculty (*N* = 8), student nurses (*N* = 12) and clinicians (*N* = 12). The demographic characteristics of participants are provided in Table 3.

**Table 3:** Demographic characteristics of participants

Variables	Nurse faculty	Clinicians	Students
Gender			
Male	1	7	4
Female	7	5	8
Age group			
21–30 years	1	1	12
31–40 years	5	11	–
41–50 years	1	–	–
51 years and above	1	–	–
Level of education			
Diploma	–	–	
First degree	–	8	
Masters degree	8	4	
Doctorate degree	–	–	
Educational level discipline			
BEd health sciences	1	–	
General Nursing	–	–	
B.Sc Nursing	2	7	
M.Sc Nursing	–	4	
Mphil Nursing	2	–	
MPH	2	–	
Others	1	1	
Rank/Position			
Senior nurse faculty	2	–	
Junior nurse faculty	6	–	
DDNS	–	4	
Principal nursing officer	–	8	
Senior nursing officer	–	–	
Nursing Officer	–	–	
Senior staff nurse	–	–	
Staff nurse	–	–	
Number of years of work			
3–5 years	6	1	
6–8 years	–	9	
9–11 years	1	2	
12 years and above	1	–	

**Figure 1:**
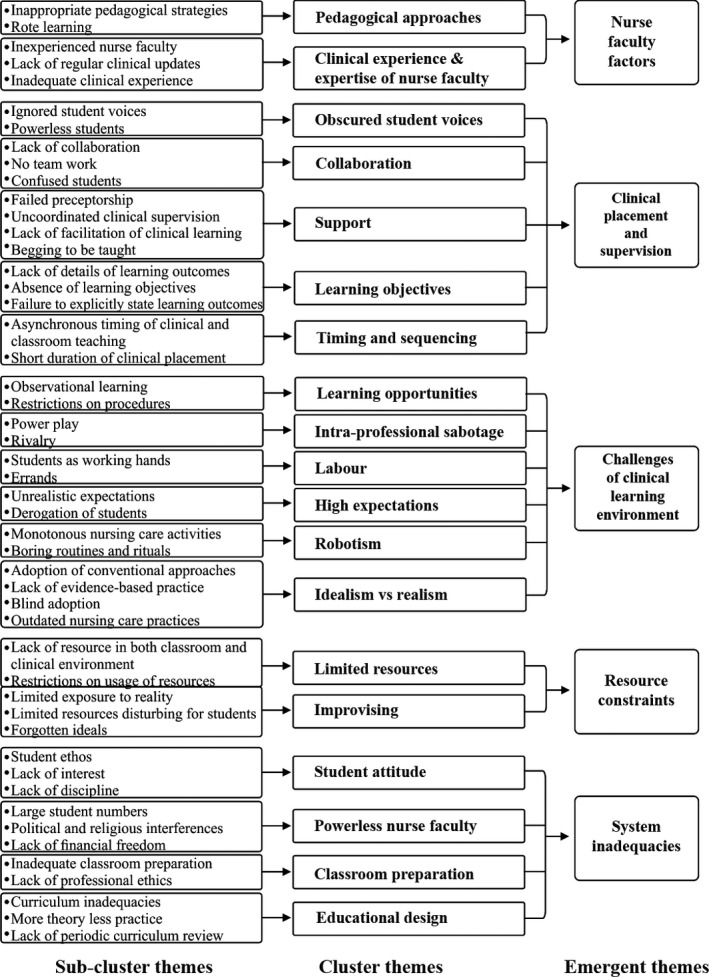
The final thematic map

Five emergent themes were identified in this study: system inadequacies, resource constraints, challenges of the clinical learning environment, clinical placement and supervision and nurse faculty factors. The overall thematic map for this study is illustrated in Figure 1.

### 4.1 System inadequacies

The existence of the TPG in this setting revolved around inadequacies of fundamental issues of nursing education and practice. Participants felt that the educational design and implementation of nursing education programmes did not promote practical skills acquisition and preparation of the nursing student to face the realities of clinical nursing practice. The relative systemic inadequacy was highlighted in the observations of a student comparing her comptency to other students also studying nursing in Germany while they were on clinical placement in the same unit:

“…Our colleagues from Ghana here, they went to Germany to study nursing and they are that competent? They are that good? So it has to actually and seriously do with the training here” [Student]

Students at site B largely believed that the curriculum adopted for the education of degree nurses was not designed to adequately promote theory‐practice integration:

“It is the curriculum that does not favour us, because they feature in more of the theory than the practical …” [Student].

Challenges with curriculum implementation, rather than adequacy for theory‐practice integration, were perceived by some nurse faculty as the main issue contributing to the TPG:

“... in terms of the curriculum that we work with in the educational institutions, it is adequate, you know, enough such that if everything had been done according to the curriculum, we should expect the practice to also be adequate” [Nurse faculty].

The department of nursing had little control over the number of student nurses admitted and blamed the situation on political and religious inteferences:

“There are certain lapses in terms of the numbers (of students admitted) which to some extent will not be our fault because we do not determine what numbers are admitted” [Nurse faculty]

Student nurse‐to‐nurse faculty/clinician ratio increases with large admissions and reduces the learning opportunities in the classroom, simulation rooms and clinical environment:

“... in the skills lab, for you to do effective skills training, the numbers need to be smaller. The students need to have hands‐on training. But then, because of the huge numbers, most students become passive… they just stand and watch, hoping that just by watching, they have learnt the skills like that but that is not possible” [Nurse faculty]

Coupled with large student numbers, faculty and clinicians both observed a decline in the enthusiam of student nurses towards learning and attributed the observation to the brighter job prospect associated with nursing as the main deciding factor in choosing a career rather than the passion to care:

"It is about their attitude on the ward, when they come they are not interested in learning, they are interested for the day to just fly by and they will go back to the school" [Clinician]

“I see contemporary nursing in Ghana to be an issue of “I am looking for a job…” [Nurse faculty]

### 4.2 Resource constraints

The general lack of resources, in an atmosphere of systemic inadequacies, was another main contributor to the TPG. This had pervasive implications on the activities of nurse faculty, clinicians and students in terms of promoting theory‐practice integration. One clinician described the magnitude of the problem with a feeling of hopelessness:

“Like it or not, we are not going to get to a situation whereby you will have all the resources to be able to make a bed, a suitable simple bed for a patient to be admitted into” [Clinician]

Aside the limited number of nurse faculty and clinicians, simulation rooms and clinical environment also lacked equipment and supplies needed to effectively demonstrate concepts and procedures:

“… our demonstration room or skills lab … we don't even have a single dummy so if you're talking about turning of patient, if we are talking about giving injection, you don't have anything to demonstrate. So it's just theory, theory, theory…” [Nurse faculty]

To navigate clinical procedures, unavailable equipment or supplies had to be improvised. The situation of improvising for almost everything prevented students from experiencing the realities of procedures or skills with the ideal equipment or supplies:

“… practice makes you perfect, it can either perfect you or make you get used to a bad habit. So if you are always used to improvising and not following the right protocol because you have to manage, because there are no resources, at the end, you come out (from training) and you forget the right way you have to use to carry out a nursing procedure ” [Student]

### 4.3 Challenges of the clinical learning environment

A combination of system inadequacies and resource constraints helped in introducing challenges for student nurses attempting to acquire practical skills and to experience the realities of patient care.

Clinicians often adopted unconventional and simple approaches to clinical procedures and patient care activities. These were usually not in consonance with textbook dictates:

“You learn the right thing in class… but you come to the clinical area, the equipment are not even there, what will you do? You get used to it (unconventional approach) because you have been practicing like that (unconventionally)… when you come out (of training) there's no way you can know the right thing…” [Student]

The blind adoption of practices from other cultures and settings, without any modification to suit the context and patient needs, was identified as one factor responsible for the disharmony between theory and practice:

“…we are relying on what people have written about those things in their setting and the context is different… so when they (students) get there (clinical setting), it is not going to be the same, so you will see it as a gap” [Nurse faculty]

Students also observed that clinical activities were routine, ritualistic and monotonous, causing students to become uninterested and apathetic in clinical learning activities:

“ …we go (to the clinical setting) it is one way, you are always following vital signs, medication… it is just a one way thing we keep doing" [Student]

Another challenge which confronted students on clinical placement was the high expectations clinicians held of them regarding their competency. Students were perceived as additional working hands and expected to assume full duties like regular staff rather than students who needed to learn:

“When you come to the ward, they treat you like you are part of the staff, you are expected to work … it is just that you do not get paid, that is the only difference” [Student]

Students also reported clinicians using them to accomplish personal and non‐clinical tasks such as running of errands during clinical placement:

“…he will just tell you go and buy food … it is more or less like it's a responsibility we are just here to do. It is not like we are learning…” [Student]

There appeared to be a power play and rivalry between university undergraduate students and graduates from NTCs, who formed the majority of nurses in the clinical environment. This rivalry is thought to arise from the relatively higher ranking the undergraduates attain immediately after completion. Graduates of the NTCs stereotyped the university students as practically inept and arrogant and hence were unwilling to assist their clinical learning:

“… there's that kind of rivalry … when it comes to the staff nurses, those who have just finished their diploma and the certificate, I think they have that rivalry with the degree students … you can calm yourself down as much as possible but some will still not teach you…” [Student]

"… if there is any power it seems we will be in‐charges over them. So that kind of power rivalry also exists and it impedes the learning of the practical aspect" [Student]

### 4.4 Clinical placement and supervision

The organization of activities of clinical placement and supervision of students also had challenges that contributed to the widening of the TPG. Learning objectives and outcomes during a clinical placement were perceived as vague; lacking in detail and explicitness and absent in some instances:

“Most of them come to the hospital and they do not even know what they are coming to learn, some schools do not accompany (students with) any competencies to the hospitals” [Clinician]

“As third year students offering BSc nursing, I want for you to set objectives for your clinical period, if you set out objectives, meet me anywhere, let us discuss your objectives and let us see how we can meet the objectives you have set. Before you come to the clinical area, you need to have a set of objectives. …if you do not have objectives then you are just coming and going and you may not achieve anything. And not even a single one (student) was ready” [Clinicians]

Clinical placements were not organized to be in synchrony with theoretical learning activities in the classroom. As a result of lack of space and/or poor organization, students had to endure placement in clinical units that were not directly related to the content discussed in the classroom prior to the placement:

“…we don't actually in real sense practice what we studied that trimester because when we come we are many so they have to divide us, everybody go here, this go there… so even what we really studied in the class we don't get to practice it at all” [Student]

Students also felt the time allocated for clinical placement was always too short to follow interesting clinical cases and to promote clinical learning:

“… our clinical system is not all that good, because we go to the hospital once a week … you go this week, you meet a client with a condition, the following week by the time you go (to the hospital) the client has been discharged” [Student]

Although both faculty and clinicians identified preceptorship as the approach adopted by both sites to provide support for students and to help bridge the TPG, students’ narrations neither confirmed any interaction with preceptors nor their existence:

Moderator “Are you assigned to preceptors?”

Student No no

Moderator Do preceptors handle you when you come on clinicals?

Student No no no. They just assign you, you are suppose to go to male ward, you just go there! At times if you go and you are not even the one looking for the in‐charge, you will even finish the clinicals and the in‐charge won't know”

“The main thing is the fact that we teach the students, we sit back and say go” [Nurse faculty]

The preceptorship system was ineffective because of the lack of preceptor training and the multiple roles preceptors assumed. Clinicians who participated in this study were mostly ward managers and also doubled as preceptors based on their own initiative. There was no formal engagement between the preceptor group and any educational institution. The responsibility to facilitate clinical learning was therefore perceived as personal and discretionary by clinicians:

“As clinicians whatever you do (to promote student learning), you are doing it on your own volition. I am paid to nurse clients. I am not paid to teach students” [Clinician]

Students had to establish personal relationships with clinicians to facilitate their own clinical learning:

“It is more or less like it depends on the individual student's rapport he establishes with the nurse that indicate that he will teach you or not” [Student]

### 4.5 Nurse faculty factors

Some clinicians raised concerns regarding the clinical experience and expertise of nurse faculty suggesting that most nurse faculty were either inexperienced or had lost touch with the realities of nursing practice due to prolonged absence from active clinical work:

“… you finished, whether university or nursing training college and you go straight into the classroom, there is no doubt about the fact that you cannot be a better teacher than somebody who would have practiced for four or five years” [Clinician]

“…maybe it is a long time some of the educators have stepped foot in the wards, they do not know what is happening in the wards and so what do they teach the students in the school?” [Clinician]

Pedagogical approaches used by some nurse faculty members promoted rote learning and was devoid of innovations to facilitate critical thinking and problem‐solving skills. This approach contributed to the students’ seeming lack of interest in learning since assessment and evaluation predominantly centred on recognition and recall:

“You go and read on the internet, read the books and come and pour (regurgitate) to them and they are forced to chew (memorize) and so you will find them thinking that there is no need to go to the ward because I can chew (memorize), the lecturer has given us a handout, so I will chew (memorize) and give it back (regurgitate) to them” [Clinician]

“ … lecturers have to adopt more practical ways of teaching. We should shift more and more away from the theory kind of thing and do practical teaching” [Nurse faculty]

## 5 DISCUSSION

The recognition of the complexity of healthcare delivery systems and the need for parallel improvements in nursing roles prompted the introduction of major reforms in nursing education in some parts of the world (Marrow, 2009; Rich & Nugent, 2010; Salminen et al., 2010; Spitzer & Perrenoud, 2006a). These reforms were based on an understanding of the TPG and were intended, among other things, to help bridge the gap between theory and practice. One of the many reforms included the shift in nursing education from the traditional hospital‐based training into higher education institutions. Experiences from these regions showed that rapid integration of nursing programmes into the higher educational institutions had a negative impact on: (a) nursing faculty members adjustment to their new roles in the higher educational settings; (b) student competencies and (c) content and structure of the nursing curriculum (Spitzer & Perrenoud, 2006a, 2006b ). Findings from this study suggest nursing education in the research setting may be undergoing a similar transition.

The powerlessness of faculty identified in this study may be related to the low academic qualifications and preparedness of nursing faculty for new roles in the university settings as observed in other regions (Jackson & Butterworth, 2007; Spitzer & Perrenoud, 2006b; Turale, Ito, & Nakao, 2008). Only one faculty member at the department of nursing of the site held a doctoral level qualification at the time of writing. Most universities in the research setting are now offering baccalaureate nursing programmes without any clear road map to guide the process of integration of nursing education into these higher education institutions. In settings where nurse faculty qualifications are not major issues, political interferences in nursing education activities usually occur at the national level as part of wider healthcare reforms (Rich & Nugent, 2010). The religious and political interferences cited in this study occurred at the level of the university.

The design and implementation of educational programmes to achieve and maintain a thorough integration of theoretical knowledge and practical skills has been a challenge for nurse faculty and other stakeholders in nursing education (Patersen & Grandjean, 2008; Rich & Nugent 2010; Spitzer & Perrenoud, 2006a; Wynaden, Orb, McGowan, & Downie, 2000). As it may appear, a clear definition and understanding of learning outcomes is the central element of any educational design and implementation strategy. Learning activities, clinical learning environment and assessment tasks need to reflect the learning outcomes to motivate students to learn and ensure knowledge transfer (Botma, et al., 2015). The need for the alignment is further highlighted in a study by Tiwari et al., (2005) suggesting that assessment tasks rather than the curriculum determine student learning activities and interest.

Debates in the literature highlight two standpoints on the profile of an ideal graduate nurse. Academics and nurse faculty favour a graduate nurse with a broad academic profile equipped with knowledge and skills for lifelong learning such as critical thinking, relevant information searching, high level analysis and synthesis of information and decision‐making (Spitzer & Perrenoud, 2006a). Graduates with a competency‐based profile are capable of applying knowledge, understanding and skills in patient care activities and are fully prepared for roles in clinical practice. Although available evidence shows that graduates with a broad academic profile become fully competent within 3–6 months of full time clinical work, graduates with a competency‐based profile are preferred by employers, hospital administrators and clinical nurse managers (Clark, Maben, & Jones, 1997; Kelly, 1996; Spitzer & Perrenoud, 2006a). Achieving either type of graduate profile requires quality and extensive interaction within a stimulating community of learning consisting of other students, nurse faculty, preceptors, other clinicians and patients.

The curriculum widely adopted for nursing education in the reseach setting appears to focus on a competency‐based profile. However, the programme outcomes are not competency‐based outcomes. Aside personal attributes, the low level of motivation of students towards learning activities may be related to the lack of constructive alignment between learning outcomes and learning activities. Although job security and personal rewards are amongst the foremost reasons for choosing nursing as a career (Boughn, 1994, 2001 ; Miers, Rickaby, & Pollard, 2007; Williams, Wertenberger, & Gushuliak, 1997), the long‐term impact of decisions rooted in these motivations on student conduct and attitudes towards learning has not been established. Innovative pedagogical approaches promoting participatory learning may boost the attitude of prospective students attracted to the profession of nursing.

Traditional approaches such as lecturing, predominantly promote rote learning rather than critical thinking and problem‐solving (Turale et al., 2008). Problem‐based learning, compared with the traditional lecture method, has been shown to be a more effective approach in increasing the level of knowledge and attitudes of students towards learning (Arthur, 2001; Dehkordi & Heydarnejad, 2008; Hwang & Jang, 2004).

As the totality of findings of this study have suggested, participants had no prior, conscious knowledge and understanding of the existence of the TPG. This is evident, amidst the resource constraints, in the poor attempt at establishing a community of learning with a shared mental model of learning outcomes and learning activities and the lack of support for student learning in the clinical learning environment. In other resource‐constrained settings, nursing and medical schools have invested in electronic learning systems to simultaneously provide greater educational opportunities for students and to enhance faculty effectiveness and efficiency (Frehywot et al., 2013). This appears not to be the case in the research setting.

The tendency of staff nurses and preceptors to focus almost exclusively on patient care to the neglect of the learning needs of nursing students has been reported in the literature (Dahlke & Hannesson, 2016; Öhrling & Hallberg, 2000; Papathanasiou, Tsaras, & Sarafis, 2014; Ryan‐Nicholls, 2004). The preceptorship group in this study was an initiative of the ward managers of the hospital and therefore had no formal relationship with the university. Despite working in a teaching hospital and having a professional obligation to support the clinical learning of student nurses (Ghana Health Service [GHS], 2006), preceptors and staff nurses in this study perceived patient care as their (preceptors and staff nurses) prime responsibility. This was largely due to the lack of recognition and appreciation for supporting student learning from the nursing school or the university. Preceptors can feel motivated and appreciated in several ways including receiving positive feedback from students, issuance of a letter of appreciation from the nursing school and opportunity for participation in a professional development activity related to their area of practice (DeWolfe, Laschinger, & Perkin, 2010).

On the the element of intraprofessional sabotage, it has been argued that the lack of clarity on the scope of practice of nursing team members has the potential to cause intra‐professional workplace conflict (Eagar, Cowin, Gregory, & Firtko, 2010). Such conflicts often involve students and can affect the self‐esteem and clinical learning of the student (Algoso & Peters, 2012; O'Mara, McDonald, Gillespie, Brown, & Miles, 2014; Papathanasiou et al., 2014; Woefle & McCaffrey, 2007). Other issues which reduce the quality of clinical placements and learning and hold the potential for compromising patient safety such as holding high expectations of student nurses as if they were full time staff nurses, have been reported elsewhere (Dahlke & Hannesson, 2016; Killam & Heerschap, 2013). Poor communication and collaboration between schools of nursing and practice settings are largely accountable for the challenges related to clinical placement and learning (Dahlke & Hannesson, 2016; Killam & Heerschap, 2013; Papp, Markkanen, & Bonsdorff, 2003). A full‐time clinical coordinator, probably employed by university, may be needed to address issues of clinical sequencing, communication and collaboration and clarification of student learning objectives.

In a study designed to observe and understand the transition process from postqualification to actual nursing practice, it was suggested that the differences in ideals and values pertaining to nursing as it is being taught and the reality of nursing in practice settings (referred to as the professional‐bureaucratic work conflict), contribute to the perpetuation of the theory practice gap (Maben et al., 2006). Despite the resource constraints and the other elements of the professional‐bureaucratic work conflict such as high workloads, preceptors who participated in this study were willing to help facilitate clinical learning if they were duly recognized or compensated. It can be argued that the TPG in the research setting was largely due to the failure of stakeholders in nursing education to clearly define learning outcomes and has outcomes aligned with learning activities occurring within a stimulating and engaging community of learning. If learning outcomes are constructively aligned with learning activities which occur within a well established community of learning, the authors hold the view that the professional and bureaucratic work conflict could be minimized. Reduced conflict will provide ample opportunities for students to activate existing knowledge, engage new information, demonstrate competence and apply skills in the real world (Botma et al., 2015).

Another challenge emerging for nurse researchers and educators in the research setting was to develop culturally sensitive and appropriate nursing knowledge, theories, philosophies and ethics. Models, theories and practices adopted from foreign cultures are often poorly understood and used (Izumi, 2006; Turale et al., 2008). Educational design and implementation strategies derived from local models and practices may help promote clinical learning and practice.

### 5.1 Study limitations

Despite several attempts to ensure rigour and trustworthiness, this study had some limitations. Because of time constraint, the study was conducted in only two sites (A and B). Therefore, transferability of study findings may be limited by the choice of participants and settings. Considering that site B is a relatively new department, some of the concerns and issues identified could be related to the obvious challenges of growth as a department. Because most hospitals and nursing schools share common operational factors such as curriculum, regulatory guidelines and resource availability, findings of this study may, appear similar to the realities in other parts of the country. Since clinicians at site A interact with students from various nursing schools (including students from site B), the perceptions of clinicians may be formulated from their collective interaction with all nursing students and not only nursing students from site B. Same can be said of the experiences of the students from site B who might have experienced placements at other hospitals or clinics.

## 6 CONCLUSION

Findings of this study confirmed the existence of the TPG in the context of nursing education and practice in the country of interest in sub‐Saharan Africa and add to the growing literature acclaiming TPG as a global phenomenon. Stakeholders of nursing education and practice in the research setting are yet to realize the realities of the implications of the TPG and its associated challenges on contemporary nursing education and practice. In the context of the research setting, the existence of TPG revolved around system inadequacies; resource constraints; challenges of the clinical learning environment; clinical placement and supervision; and nurse faculty factors. Inadequate establishment of a community of learning with a shared mental model of learning outcomes aligned with learning activities and sessions was largely accountable for the theory practice gap in this setting.

## CONFLICT OF INTEREST

No conflict of interest has been declared by the authors.

## AUTHOR CONTRIBUTIONS

DAS conceived and designed the study and drafted the manuscript. DAS and MAS collected and analysed the data. JG and DAS funded the study. JG, MAS and DAS made critical revisions on the paper. JG and JN supervised the study.

All authors have agreed on the final version and meet at least one of the following criteria [recommended by the ICMJE (https://www.icmje.org/recommendations/ )]:

- substantial contributions to conception and design, acquisition of data or analysis and interpretation of data;
- drafting the article or revising it critically for important intellectual content.
